# Genome-wide analysis of the response to nitric oxide in uropathogenic *Escherichia coli* CFT073

**DOI:** 10.1099/mgen.0.000031

**Published:** 2015-10-13

**Authors:** Heer H. Mehta, Yuxuan Liu, Michael Q. Zhang, Stephen Spiro

**Affiliations:** ^1^​ Department of Biological Sciences, University of Texas at Dallas, 800 W Campbell Road, Richardson, TX 75080, USA; ^2^​ Center for Systems Biology, University of Texas at Dallas, 800 W Campbell Road, Richardson, TX 75080, USA

**Keywords:** ChIP-seq, nitric oxide, NsrR, RNA-seq, uropathogenic *E. coli*

## Abstract

Uropathogenic *Escherchia coli* (UPEC) is the causative agent of urinary tract infections. Nitric oxide (NO) is a toxic water-soluble gas that is encountered by UPEC in the urinary tract. Therefore, UPEC probably requires mechanisms to detoxify NO in the host environment. Thus far, flavohaemoglobin (Hmp), an NO denitrosylase, is the only demonstrated NO detoxification system in UPEC. Here we show that, in *E. coli* strain CFT073, the NADH-dependent NO reductase flavorubredoxin (FlRd) also plays a major role in NO scavenging. We generated a mutant that lacks all known and candidate NO detoxification pathways (Hmp, FlRd and the respiratory nitrite reductase, NrfA). When grown and assayed anaerobically, this mutant expresses an NO-inducible NO scavenging activity, pointing to the existence of a novel detoxification mechanism. Expression of this activity is inducible by both NO and nitrate, and the enzyme is membrane-associated. Genome-wide transcriptional profiling of UPEC grown under anaerobic conditions in the presence of nitrate (as a source of NO) highlighted various aspects of the response of the pathogen to nitrate and NO. Several virulence-associated genes are upregulated, suggesting that host-derived NO is a potential regulator of UPEC virulence. Chromatin immunoprecipitation and sequencing was used to evaluate the NsrR regulon in CFT073. We identified 49 NsrR binding sites in promoter regions in the CFT073 genome, 29 of which were not previously identified in *E. coli* K-12. NsrR may regulate some CFT073 genes that do not have homologues in *E. coli* K-12.

## Data Summary

1. RNA-seq data have been deposited in the GEO database; accession number: GSE69830 (url – http://www.ncbi.nlm.nih.gov/geo/query/acc.cgi?acc = GSE69830)
2. ChIP-seq data have been deposited in the GEO database; accession number: GSE69829 (url – http://www.ncbi.nlm.nih.gov/geo/query/acc.cgi?acc = GSE69829)

## Impact Statement

Uropathogenic *Escherichia coli* (UPEC) is one of the leading causes of urinary tract infections in humans. Processes facilitating survival of the pathogen in the host are not fully understood. Nitric oxide (NO) is generated by host immune cells as a defence mechanism, and NO scavenging enzymes are probably needed by UPEC to survive in the host environment. Understanding the NO sensing and detoxification mechanisms of UPEC will help to further understand its interaction with the host. The present data suggest that exposure to NO causes a reprogramming of energy metabolism in UPEC, and may contribute to increased expression of virulence-associated genes (including NO scavenging enzymes). Thus, virulence determinants may be expressed by UPEC in response to a host-generated signal, and NO may act as a signal of a suitable host environment.

## Introduction

Extraintestinal *Escherichia coli* are a group of bacteria that can survive as harmless human intestinal inhabitants but are serious pathogens when they enter the appropriate environment (Welch *et al.*, 2002). Uropathogenic *Escherichia coli* (UPEC) strain CFT073 is one such pathogen that is a causative agent of urinary tract infections (UTIs) and was isolated from the blood of a woman suffering from acute pyelonephritis (Mobley *et al.*, 1990). UPEC is responsible for 80 % of all symptomatic and asymptomatic UTIs (Roos *et al.*, 2006). During UTI, the mucosal inflammatory response is activated, which causes neutrophils to infiltrate and migrate through the tissues and into the urine (Godaly *et al.*, 2001). Thus, UPEC is exposed to the defence mechanisms of the innate immune system.

The antimicrobial properties of nitric oxide (NO) are exploited by cells of the innate immune system. In response to stimulation by proinflammatory cytokines and lipopolysaccharides of microbial pathogens, phagocytic cells express an inducible nitric oxide synthase (iNOS), which oxidizes arginine to produce NO (Fang, 1997; Fang & Vazquez-Torres, 2002; Mowat *et al.*, 2010). iNOS is also expressed in epithelial cells of the urinary tract (Poljakovic *et al.*, 2001). NO (and related N radicals that are derived from NO) target proteins containing iron–sulfur clusters, haem and thiols (Fang, 1997; Fang & Vazquez-Torres, 2002; Kim *et al.*, 1995; Ren *et al.*, 2008). Because of the cytotoxic properties of NO and its congeners, bacteria that proliferate within the host often employ strategies to convert NO into a non-toxic product. *E. coli* has three known enzymes that detoxify NO. Flavohaemoglobin (Hmp) is an NO denitrosylase that oxidizes NO to nitrate, and may reduce NO to N_2_O in the absence of oxygen (Gardner & Gardner, 2002; Hausladen *et al.*, 2001). Hmp has a role in protecting UPEC from NO stress in the host environment (Svensson *et al.*, 2010). Flavorubredoxin (FlRd or NorV) together with its NADH-linked reductase NorW reduces NO to N_2_O (Gomes *et al.*, 2000). FlRd is sensitive to oxygen *in vitro*, and so the enzyme has been described as an ‘anaerobic’ NO reductase (da Costa *et al.*, 2003; Gardner *et al.*, 2002). The periplasmic nitrite reductase Nrf can catalyse the reduction of NO to ammonia under anaerobic conditions, a reaction that might contribute to defence against NO (Poock *et al.*, 2002). Recently, it has been suggested that the hybrid cluster protein Hcp is a key player in the response to NO under anaerobic conditions, when *E. coli* is lacking all previously known NO scavenging enzymes (Cole, 2012), although the enzymic activity of Hcp remains enigmatic.

The response to NO in *E. coli* involves several transcription factors, including NsrR, FNR, SoxR, OxyR, NorR and Fur (Bodenmiller & Spiro, 2006; Cruz-Ramos *et al.*, 2002; D'Autréaux *et al.*, 2002; Gardner *et al.*, 2002; Hausladen *et al.*, 1996; Pomposiello & Demple, 2001). Most of these regulators primarily sense other signals such as oxygen, hydrogen peroxide and iron (Bodenmiller & Spiro, 2006), but NorR and NsrR serve as dedicated sensors of NO (Tucker *et al.*, 2010). The mononuclear non-haem iron centre of NorR directly senses NO, in response to which NorR activates transcription of *norVW* (D'Autréaux *et al.*, 2005; Tucker *et al.*, 2010). NsrR is an [Fe–S] protein that is an NO-sensitive repressor of its target genes (Bodenmiller & Spiro, 2006; Filenko *et al.*, 2007; Pullan *et al.*, 2007). The *hmp* gene is subject to complex regulation by multiple regulators including NsrR and FNR (Spiro, 2007). Apart from *hmp*, the NsrR regulon contains various genes implicated in the NO stress response, such as *ytfE*, *hcp* and the *nrf* operon (Tucker *et al.*, 2010). In addition to recognizing an 11–1–11 bp inverted repeat sequence in its target promoters, it has been suggested that NsrR can also bind to a single copy of the 11 bp motif (Partridge *et al.*, 2009). In *E. coli*, the only known genes activated by NorR are *norVW*, but binding sites for NorR have been found upstream of the *hmp* gene in *Pseudomonas aeruginosa*, *Pseudomonas putida* and *Vibrio cholera*e and the gene encoding a respiratory NO reductase in the denitrifier *Ralstonia eutropha* (Rodionov *et al.*, 2005; Stern *et al.*, 2012; Tucker *et al.*, 2004). Low concentrations of NO are generated endogenously by *E. coli* as a by-product of respiratory nitrate and nitrite reduction (Corker & Poole, 2003; Ji & Hollocher, 1988). Nitrate and nitrite are sensed directly by the NarXL and NarQP two-component regulatory systems (Gunsalus, 1992; Stewart, 1993). Thus, during nitrate or nitrite respiration, complex changes occur in the transcriptome that are mediated by NarXL/NarQP in addition to the above-mentioned NO-responsive regulators (Constantinidou *et al.*, 2006).

Global gene expression analysis has been used to study UPEC during UTI and demonstrates that UPEC is directly exposed to NO in the host environment (Hagan *et al.*, 2010; Haugen *et al.*, 2007; Roos & Klemm, 2006; Snyder *et al.*, 2004) as well as to nitrate in urine (Green *et al.*, 1981; Radomski *et al.*, 1978). UPEC is more resistant to the stress imposed by acidified nitrite than K-12 strains of *E. coli* (Bower & Mulvey, 2006) and may also be more resistant to a prolonged exposure to NO (Svensson *et al.*, 2006), in which case toxicity might be due to N radicals derived from NO. We have shown that CFT073 recovers from an exposure to NO no better than a K12 strain and that recovery is partly, although not entirely, dependent on Hmp (Spiro, 2011). Thus, we were motivated to undertake a deeper analysis of the determinants of NO resistance in CFT073. In this paper, we show that apart from Hmp, FlRd is a major contributor to aerobic NO detoxification in UPEC. We also show that CFT073 possesses at least one novel anaerobic NO scavenging mechanism in addition to Hmp and FlRd. We use expression analysis to examine the response of CFT073 to a physiological source of NO, and map NsrR binding sites in the CFT073 genome.

## Methods

### Bacterial strains and growth conditions

The strains used in this work are listed in Table S1 (available in the online Supplementary Material). The rich medium was L broth (per litre: 10 g tryptone, 5 g yeast extract, 5 g NaCl), supplemented with 0.5 % glucose for anaerobic cultures. To treat cultures with NO, 50 μM spermine NONOate (which releases two equivalents of NO with a half-life of 39 min at 37 °C; Cayman Chemicals) was added to cultures during the early exponential phase (OD_650_ of 0.15–0.3). [NONOate has the chemical formula R^1^R^2^N-(NO^− ^)-*N* = O.] Anaerobic cultures were grown in filled bottles supplemented with 20 mM nitrate where indicated. Cultures for RNA isolation were grown anaerobically in MOPS minimal medium (Neidhardt *et al.*, 1974) supplemented with 0.05 % Casamino acids, 0.5 % glucose (and 20 mM nitrate as indicated), and 5 μg vitamin B1 ml^− 1^. Gene deletions were made using the method of Datsenko & Wanner (2000).

### Oxygen and nitric oxide consumption assays

For oxygen consumption assays, 30 ml cultures grown aerobically in L broth (with and without 1 h of 50 μM spermine NONOate treatment) were harvested, washed and resuspended in 50 mM HEPES (pH 7.4), 100 mM NaCl, 5 mM KCl, 1 mM MgCl_2_, 1 mM NaH_2_PO_4_, 1 mM CaCl_2_ and 1 mM glucose (Stevanin *et al.*, 2000). All samples were resuspended at equal cell densities. Oxygen consumption was measured using a Clark-type oxygen electrode (Hansatech Instruments). A 100 μl aliquot of cells was added to 500 μl buffer in a capped, water-jacketed chamber at 37 °C. The NO sensitivity of oxygen uptake was measured by addition of 20 μM proli NONOate (which releases two equivalents of NO with a half-life of 1.8 s at 37 °C; Cayman Chemicals) when the oxygen concentration was 60, 120 or 180 μM. For NO consumption assays, 30 ml of cultures grown anaerobically in L broth (treated with 50 μM spermine NONOate for 2 h or grown with 20 mM nitrate) were harvested and resuspended in the same HEPES buffer (without glucose). The water-jacketed chamber housing an amperometric NO-specific electrode (ISO NOP electrode; WPI Instruments) was maintained at 37 °C. Cell suspension (0.5 ml) and buffer (1.5 ml) were added to the chamber, and oxygen was removed with 5 μl of 1 M glucose, 5 μl of 30 mg glucose oxidase ml^− 1^ and 5 μl of 7 mg catalase ml^− 1^. When oxygen was undetectable, 20 μM proli NONOate was added and the rate of NO consumption was measured. The same procedure was used to measure NO consumption by cell fractions.

### Cell fractionation

Cells were fractionated using a modification of a previously described procedure (Alefounder & Ferguson, 1980). Cultures were grown anaerobically in 300 ml L broth supplemented with 0.5 % glucose, and in some cases were treated with 50 μM spermine NONOate. Cultures were harvested by centrifugation and washed twice in 10 mM potassium phosphate buffer (pH 7.6). Cell pellets were resuspended in 5 ml spheroplasting buffer (0.5 M sucrose, 3 mM sodium EDTA and 0.1 M Tris/HCl, pH 8.0), 0.2 mg lysozyme ml^− 1^ was added and the suspension was incubated at 30 °C for 30 min. The suspension was centrifuged at 12 200 ***g*** for 15 min at 4 °C, and the supernatant (periplasmic fraction) was kept on ice. The pellet was resuspended in 1 ml of 0.1 M Tris/HCl (pH 8.0) and slowly added drop-wise into 4.5 ml water with constant stirring at 4 °C. After the mixture became homogeneous, it was centrifuged at 47 800 ***g*** for 1 h at 4 °C. The supernatant (cytoplasmic fraction) was stored on ice and the pellet (membrane fraction) was resuspended in 0.1 M Tris/HCl (pH 8.0) and kept on ice. Malate dehydrogenase was used as a marker to check the integrity of cell fractions. Malate dehydrogenase activity (Sutherland & McAlister-Henn, 1985) was detected only in cytoplasmic fractions.

### RNA sequencing

Cultures were grown in triplicate as described above, and total RNA was isolated using the Qiagen RNeasy Protect Bacteria Mini kit. For rRNA depletion, samples were treated using the MICROBExpress Bacterial mRNA Enrichment kit (Life Technologies) according to the manufacturer's instructions. Samples were cleaned with the Zymo RNA Clean and Concentrator kit (Zymo Research) and then subjected to a second cycle of rRNA depletion. RNA was recovered by ethanol precipitation. Library preparation and whole transcriptome shotgun sequencing (RNA-seq) was performed at the University of Texas Southwestern Medical Center Genomics and Microarray Core Facility.

The program Bowtie (Langmead *et al.*, 2009) was used to align RNA-seq reads to the genome of *E. coli* CFT073 (GenBank accession no. AE014075.1) with default parameters. To estimate transcript abundances, transcripts per million (TPM) values were calculated using RNA-Seq by expectation-maximization (RSEM; Li & Dewey, 2011). Gene annotations were obtained from the European Nucleotide Archive (accession no. AE014075.1). Differential expression between conditions with and without NO treatment was analysed using EBSeq (an empirical Bayes hierarchical model for inference in RNA-seq experiments; Leng *et al.*, 2013). A change greater than twofold and a false discovery rate cut-off of 0.05 were used to determine significant differential expression.

To identify functional categories of differentially expressed genes and to identify enriched pathways, we used the DAVID (database for annotation, visualization and integrated discovery) gene functional classification tool with default statistical parameters and Benjamini correction (Huang *et al.*, 2007) with an adjusted *P*-value cut-off of 0.05.

RNA-seq data have been deposited in the GEO database, accession number GSE69830 (Data Citation 1).

### Reverse transcriptase PCR (RT-PCR)

Wild-type CFT073 and 3X mutant cells were grown in triplicate as described for RNA-seq. Total RNA was isolated using the Qiagen RNeasy Protect Bacteria Mini kit, and 2 μg of total RNA was used to make cDNA using Ambion's RETROscript Reverse Transcription kit. Then, 1 μl of cDNA was used as template to amplify genes that were chosen for validation. PCR was performed using Thermo Scientific DreamTaq PCR Master Mix and 10 μl of PCR product was run on a 2 % agarose gel.

### Chromatin immunoprecipitation followed by high-throughput sequencing

Chromatin immunoprecipitation (ChIP) was performed as described previously on cultures grown aerobically in L broth to mid-exponential phase (Efromovich *et al.*, 2008). Immunoprecipitated and purified DNAs (10 ng) from three cultures of CFT073 were collected for sequencing, along with 10 ng of the input DNA as a control. Samples were sheared by sonication to within a size range of 200–600 bp. DNA fragments were treated using an Epicentre End-It DNA End Repair kit and 3′ A overhangs were added with DNA polymerase I (Klenow fragment). Adapters from the IlluminaTruSeq DNA sample preparation kit were ligated using LigaFast (Promega) and DNAs were amplified by PCR using primers provided in the IlluminaTruSeq DNA sample preparation kit and Phusion DNA polymerase (NEB). Products of the ligation reaction and PCR amplification in the range 300–400 bp were purified by 2 % agarose gel electrophoresis. DNA concentrations were measured using Qubit dsDNA HS Assay kits (Invitrogen). DNA sequencing was done on the Miseq (Illumina) platform following the manufacturer's instructions. For one replicate, a single-end reads, 60 bp run was performed. For the other two replicates, a paired-end reads, 100 bp run was performed. Sequence reads were aligned with the published *E. coli* CFT073 genome (AE014075.1) using the software package Bowtie with the parameters ‘bowtie -k 1 -X 500 -m 1’ (Langmead *et al.*, 2009). Peaks were identified using the peak finding algorithm of MACS2 (Zhang *et al.*, 2008), with default parameters.

For motif analysis, multiple Em for motif elicitation (MEME) was used to identify over-represented sequences (Bailey & Elkan, 1994). PatSer was used to search the genome for the presence of the NsrR position-specific weight matrix (PSWM) (Hertz & Stormo, 1999). A precision–recall curve was constructed to determine the optimal threshold for predicting high-quality NsrR binding sites. Precision was defined as the ratio of true positives (locations with an NsrR ChIP-seq peak and a predicted NsrR binding site) to true positives plus false positives (locations with a predicted NsrR binding site but no NsrR ChIP-seq peak). Recall was defined as the ratio of true positives divided by true positives plus false negatives (locations with an NsrR ChIP-seq peak but no NsrR predicted binding site; Myers *et al.*, 2013).

ChIP-seq data have been deposited in the GEO database, accession number GSE69829 (Data Citation 2).

## Results

### FlRd contributes to aerobic NO detoxification

The *norV* gene is absent from current annotations of the *E. coli* CFT073 genome (Welch *et al.*, 2002). However, we have resequenced the *norV* region and found a sequencing error in the published sequence that leads to a frameshift mutation. The corrected *norV* sequence encodes a protein product that is 99 % identical to FlRd (NorV) of *E. coli* K-12 (476/479 residues identical). Thus, we performed experiments to determine whether *E. coli* CFT073 expresses an active FlRd, and to assess its contribution to NO detoxification.

The *E. coli* K-12 FlRd functions as an ‘anaerobic’ NO reductase, although it is capable of reducing NO *in vivo* in microaerobic cultures growing under an atmosphere containing ∼5 μM oxygen (Gardner *et al.*, 2002). FlRd can also function as an oxygen reductase, albeit with a rather low affinity for oxygen (Gomes *et al.*, 2002). Enzymes from the flavo-diiron family in other organisms are inactivated by oxygen *in vitro* (Silaghi-Dumitrescu *et al.*, 2003, 2005), so a view has emerged that FlRd functions in NO detoxification only in cultures growing anaerobically. However, the *norVW* genes can be induced by sources of NO in aerobic cultures, so a physiological role for FlRd under aerobic conditions cannot be excluded (Hutchings *et al.*, 2002; Mukhopadhyay *et al.*, 2004).

**Fig. 1.**
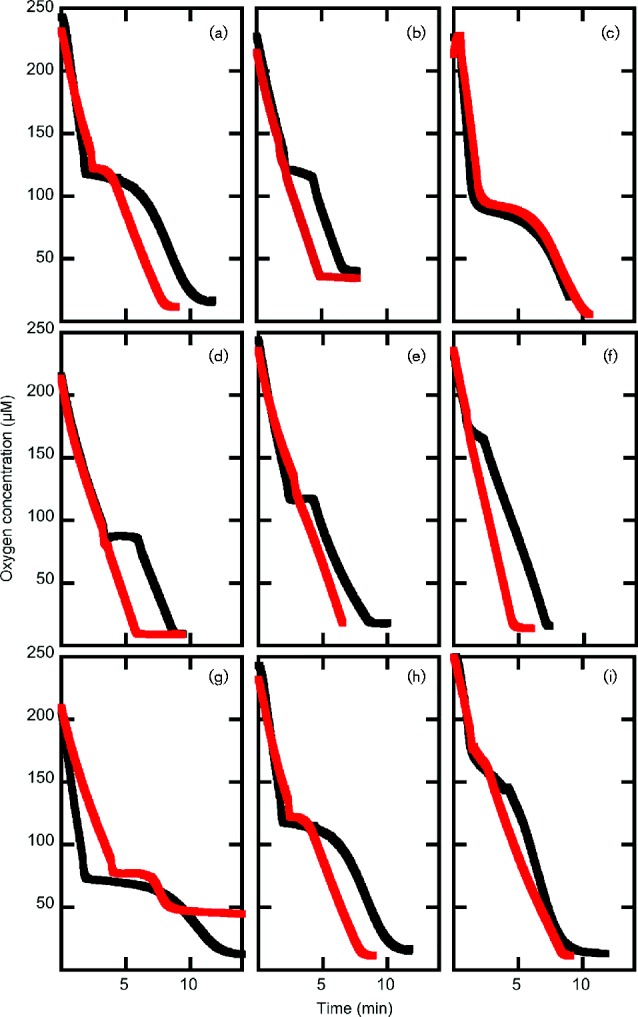
Oxygen consumption by UPEC strains grown without (black) or with (red) exposure to NO (50 μM spermine NONOate). NO (20 μM proli NONOate) was added when the oxygen concentration reached 100–120 μM to assay NO-mediated inhibition of respiration in: (a) UTD635 (Δ*hmp*), (b) UTD680 (Δ*norVW*) and (c) UTD681 (Δ*hmp* Δ*norVW*). NO-mediated inhibition of respiration was measured in wild-type (d–f) and UTD635 (Δ*hmp*, g–i) at 80 μM O_2_ (d and g), 120 μM O_2_ (e and h), and 180 μM O_2_ (f and i). The increasing resistance of uninduced cells at higher O_2_ concentrations probably reflects direct reaction of NO with O_2_.

Respiration in *E. coli* is sensitive to NO (Yu *et al.*, 1997) and the activity of an NO scavenging enzyme can be studied by observing its ability to protect aerobic respiration from NO inhibition (Stevanin *et al.*, 2000). Washed cell suspensions respiring oxygen were exposed to NO in a Clark-type oxygen electrode, and the inhibitory effect of NO was observed as a transient decrease in the rate of oxygen consumption. In these experiments, oxygen uptake returns to normal after an interval that depends upon the ability of the strain to scavenge NO (Fig. 1). Wild-type cells that were exposed to NO during growth exhibited NO-resistant oxygen uptake, while the respiration of cells that were not pre-exposed to NO was sensitive to NO (duration of inhibition 1.9 ± 0.04 min, Fig. 1e). In an *hmp* mutant, respiration became more resistant to NO if the culture was pre-induced with NO (4.3 ± 0.7 versus 1.4 ± 0.4 min inhibition for uninduced and induced cells, respectively; Fig. 1a). Respiration of the *norVW* mutant was completely NO-resistant if cells were induced with NO, but NO-sensitive (1.7 ± 0.6 min inhibition) if cells were not induced (Fig. 1b). Thus, both *hmp* and *norVW* mutants show evidence of an NO-inducible scavenging activity, which is, presumably, FlRd and Hmp, respectively. Accordingly, in an *hmp norVW* double mutant (Fig. 1c), oxygen consumption was equally sensitive to NO whether or not the cultures were exposed to NO (6 ± 0.1 and 6.3 ± 0.3 min inhibition for uninduced and induced cells, respectively). Under the growth and assay conditions used for these experiments, NO-inducible NO scavenging by CFT073 can be entirely accounted for by the combined activities of Hmp and FlRd.

By measuring NO inhibition of respiration of the *hmp* mutant at different oxygen concentrations, we concluded that FlRd can scavenge NO *in vivo* in the presence of as much as 180 μM oxygen (Fig. 1g–i). Complementing the *hmp norV* double mutant with *norV* on a plasmid expressed from an inducible promoter showed that FlRd-mediated protection of respiration was restored (data not shown). Interestingly, complementation failed if *norV* was expressed on a plasmid from its own promoter, as we have observed before (Hutchings *et al.*, 2002), and successful complementation required expression from a heterologous promoter.

### A novel inducible anaerobic NO scavenging activity

**Fig. 2.**
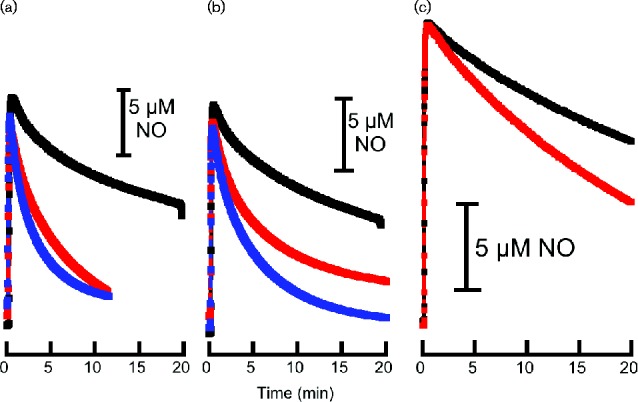
NO consumption by whole cells and membrane fractions of UPEC strain CFT073 and UTD692 (Δ*hmp* Δ*nrfA* Δ*norVW*). Cultures were grown under anaerobic conditions and induced with NO (50 μM spermine NONOate) or 20 mM nitrate. NO consumption by whole cells of (a) CFT073 and (b) UTD692, and (c) membrane fractions of UTD692 was measured using an NO electrode, in the absence of oxygen. The assay was initiated by the addition of 20 μM proli NONOate. Black: untreated cultures; red: NO-treated cultures; blue: cultures grown with nitrate.

**Fig. 3.**
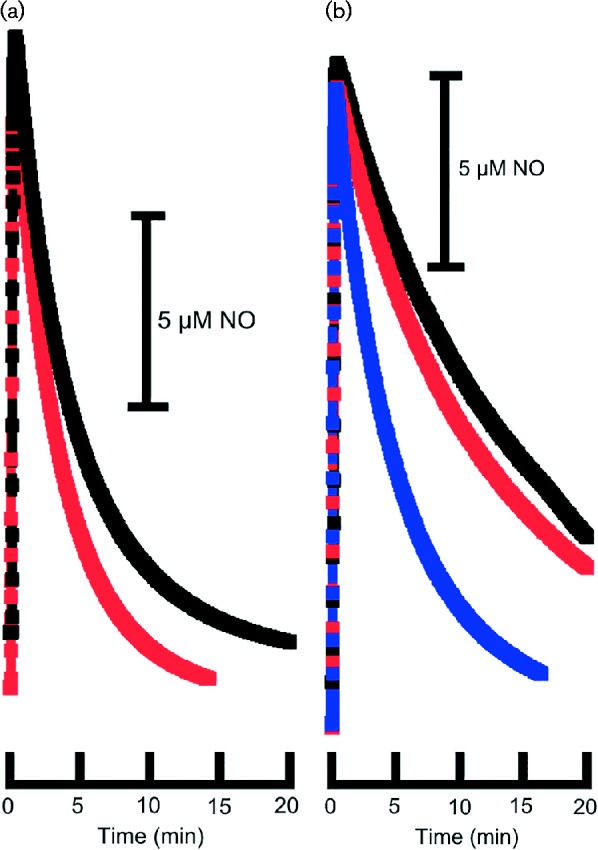
NO consumption by UPEC strains (a) UTD783 (Δ*hmp* Δ*nrfA* Δ*norVW fnr*::*kan*) and (b) UTD717 (Δ*hmp* Δ*nrfA* Δ*norVW nsrR*::*kan*). Cultures were grown under anaerobic conditions with 20 mM nitrate, or were induced with NO (50 μM spermine NONOate). The assay was initiated by the addition of 20 μM proli NONOate and the NO concentration was measured using an NO electrode in a cell suspension from which oxygen had been removed. Black: untreated cultures; red: NO-treated cultures; blue: cultures grown with nitrate.

We generated a triple mutant of *E. coli* CFT073 lacking the three known NO detoxification systems, Hmp, FlRd and NrfA (this strain is designated UTD692, and will be referred to as ‘3X’). Washed cells of the 3X strain grown anaerobically with nitrate (nitrate provides a source of endogenously generated NO under anaerobic conditions; Ji & Hollocher, 1988), or induced anaerobically with NO showed a higher rate of NO consumption compared with an uninduced strain (Fig. 2). Thus, in cells grown and assayed anaerobically, there is evidence of a novel NO-inducible activity. A wild-type strain grown and assayed under similar conditions showed only moderately increased rates of NO consumption compared with the 3X mutant (Fig. 2). The dominant activity of Hmp requires molecular oxygen, so Hmp is not expected to contribute to NO consumption under these assay conditions. The measured activity is therefore a combination of FlRd and the novel activity, the latter seeming to be a major contributor in cells grown and assayed anaerobically. Cell fractionation experiments revealed this activity to be associated with the cell membrane (Fig. 2c). Interestingly, NO uptake by membrane fractions of the triple mutant required neither an exogenous reductant nor an oxidizing agent. Possible candidates for the source of this activity include NirB (Vine & Cole, 2011b) and the hybrid cluster protein, Hcp (Cole, 2012; Vine & Cole, 2011a), although in both cases NO reduction would be dependent on NADH. Introduction of *nirB* and *hcp*-*hcr* mutations into the 3X mutant, either individually or in combination, had no effect on the NO scavenging activity, so NirB and Hcp can be excluded as the source of the activity we observe. Introduction of a *mobA* mutation (which eliminates nitrate reductase activity) into the 3X mutant eliminated the response to nitrate, confirming that nitrate reduction and therefore endogenous NO generation is probably required for nitrate-mediated induction of the NO scavenging activity. In a 3X *nsrR* mutant, NO scavenging could be induced by nitrate but not by NO in anaerobic cultures (Fig. 3). The same outcome was seen in a CFT073 *nsrR* mutant (data not shown), which further demonstrates that under these conditions, Hmp, which is de-repressed in an *nsrR* mutant (Filenko *et al.*, 2007), is not functional. In the 3X strain with an *fnr* mutation, the NO scavenging activity became constitutive (Fig. 3), implying that FNR acts negatively on the expression of the gene(s) encoding the activity. In a 3X *narL narP* mutant, the activity could be induced by nitrate but not NO in anaerobic cultures (data not shown). Individual 3X *narL* and 3X *narP* mutants behaved like the 3X parent strain.

In further attempts to identify the source of the NO scavenging activity, we tested candidate genes by introducing the corresponding deletion mutations into the 3X mutant. Candidates were identified on the basis of one or more of the following criteria: (1) a primary structure suggesting a possible role in NO metabolism; (2) a previously described expression pattern matching our observations described above; and (3) an expression pattern in our RNA-seq data (see below) similar to the behaviour of the novel activity. Some genes were also tested that might be indirectly involved in expression of this activity (e.g. *mobA*, required for the activity of enzymes containing the molybdopterin guanine dinucleotide cofactor). In this way, we showed that 26 genes or operons are not required for expression of the novel NO scavenging activity (*aegA*, *betA*, *cydAB*, *cyoAB*, *fdhE*, *hcp*-*hcr*, *mobA*, *ndh*, *nirB*, *nrdA*, *poxB*, *putA*, *rnr*, *sdhA*, *tehAB*, *yceJI*, *ydcX*, *ydhXV*, *yeaR*-*yoaG*, *yebE*, *yedY*, *yeiH*, *ygbA*, *yhaM*, *yibIH*, *ytfE*).

Further efforts to identify the source of the novel NO scavenging activity described in this paper have so far proved unsuccessful. Vine & Cole (2011b) have reported that a *norVW nrf nirB hmp* quadruple mutant of *E. coli* K-12 consumes NO at rates comparable to those of the wild-type strain. Our data suggest that Hmp and FlRd are the major NO scavenging activities of *E. coli* CFT073 under aerobic conditions, and the novel activity we describe makes a significant contribution to NO consumption only under anaerobic growth conditions.

### Transcriptional profiling

We used transcriptomics (RNA-seq) to explore the response of *E. coli* CFT073 to a source of NO. In part, this experiment was motivated by a desire to identify the gene(s) encoding the novel NO scavenging activity. Therefore, we used a strain and growth conditions identical to those used for the initial detection of this activity, as described above. That is, the transcriptome of the triple mutant was analysed in anaerobic cultures grown in the presence and absence of nitrate. By differential gene expression analysis of the RNA-seq data, we identified 525 upregulated and 649 downregulated genes in the nitrate-treated cultures (genes showing greater than twofold change with a 0.05 false discovery rate cut-off). Among the most highly upregulated genes (Table 1) were some that are known to be regulated by NsrR and to respond to NO, including *yeaR*, *ytfE*, *hcp-hcr* and *ygbA* (Filenko *et al.*, 2007; Pullan *et al.*, 2007). Previous transcriptomics experiments with *E. coli* K-12 have also shown that members of the NsrR regulon are de-repressed in cultures grown anaerobically with nitrate (Constantinidou *et al.*, 2006).

**Table 1.** Fifty genes most highly upregulated in RNA-seq data

Gene name	Locus tag	Function	Fold change^*^
*hlyC*	c3569	Haemolysin C	253.48
*ndk*	c3041	Nucleoside diphosphate kinase	201.23
*yoaG*	c2202	Hypothetical protein YoaG	167.79
*yeaR*	c2204	Hypothetical protein YeaR	160.21
*c2203*	c2203	Hypothetical protein	147.10
*c2201*	c2201	Hypothetical protein	142.23
*ytfE*	c5308	Hypothetical protein YtfE	69.63
*hcp*	c1006	Prismane protein homologue, Hcp	43.72
*c1005*	c1005	NADH oxidoreductase Hcr	37.18
*c1905*	c1905	Hypothetical protein	32.09
*cyoA*	c0543	Ubiquinol oxidase polypeptide II precursor	27.78
*fdnG*	c5623	Formate dehydrogenase, nitrate-inducible, major subunit	25.56
*cyoC*	c0541	Cytochrome *o* ubiquinol oxidase subunit III	20.18
*cyoB*	c0542	Ubiquinol oxidase polypeptide I	18.76
*proV*	c3230	Glycine betaine/l-proline transport ATP-binding protein ProV	18.00
*c3158*	c3158	Putative tail component of prophage	17.82
*c0938*	c0938	Hypothetical protein	17.55
*proW*	c3231	Glycine betaine/l-proline transport system permease protein ProW	17.06
*glnK*	c0568	Nitrogen regulatory protein P-II 2	16.11
*c2852*	c2852	Pseudogene	15.47
*c0955*	c0955	Probable phage tail protein	15.42
*cspB*	c3184	Cold shock-like protein CspB	15.40
*c0966*	c0966	Putative phage tail protein	14.80
*ompF*	c1071	Outer-membrane protein F precursor	13.71
*c0936*	c0936	Hypothetical protein	13.08
*c0740*	c0740	Hypothetical protein	12.95
*cyoD*	c0540	Cytochrome *o* ubiquinol oxidase protein CyoD	12.89
*c3192*	c3192	Unknown protein encoded by cryptic prophage	12.77
*yfdR*	c3202	Hypothetical protein YfdR	12.75
*yfdN*	c3191	Hypothetical protein YfdN	12.61
*c0939*	c0939	Hypothetical protein	12.57
*matB*	c0404	Hypothetical protein YagZ precursor	12.53
*wcaF*	c2580	Putative colanic acid biosynthesis acetyltransferase WcaF	12.33
*c1870*	c1870	Hypothetical protein YdcX	11.85
*gltK*	c0737	Glutamate/aspartate transport system permease protein GltK	11.74
*dppB*	c4358	Dipeptide transport system permease protein DppB	11.50
*sdhD*	c0800	Succinate dehydrogenase hydrophobic membrane anchor protein	11.21
*c0937*	c0937	Hypothetical protein	11.10
*c0940*	c0940	Hypothetical protein YbiI	10.67
*c0941*	c0941	DNA adenine methylase	10.58
*fimB*	c5391	Type 1 fimbriae Regulatory protein FimB	10.37
*c0569*	c0569	Hypothetical protein	10.33
*c4376*	c4376	Hypothetical protein	10.22
*yagY*	c0403	Hypothetical protein YagY precursor	10.21
*motA*	c2305	Chemotaxis MotA protein	10.00
*uhpT*	c4590	Hexose phosphate transport protein	9.91
*c0897*	c0897	Hypothetical protein	9.74
*c0963*	c0963	Putative phage baseplate assembly protein	9.55
*wcaE*	c2581	Putative colanic acid biosynthesis glycosyltransferase WcaE	9.48
*yhdT*	c4022	Hypothetical protein YhdT	9.42

* The CFT073 3X mutant was cultured anaerobically in the presence and absence of nitrate, as described in the text. Fold change is the ratio of expression levels in the presence and absence of nitrate, and is the posterior fold change (the fold change computed from normalized data), calculated by EBSeq.

RNA was extracted from anaerobically grown cultures, so it was surprising that the most highly upregulated genes included some involved in oxidative phosphorylation (*cyoABCDE*, *sdhABCD* and *nuoEF*) and the tricarboxylic acid (TCA) cycle (*acnB*, *icdA*, *sucD*, *lpdA*, *sdhABCD*, *fumA* and *mdh*). Several genes encoding ABC transporters (*dpp* operon, *proVW*, *gltK*, *kpsMT*) were also upregulated. Other genes showing increased expression in nitrate-grown cells were those involved in nitrogen metabolism (nitrate respiration), DNA repair and the SOS response, bacterial motility and chemotaxis. The most highly downregulated genes included some encoding enzymes involved in anaerobic metabolism, including hydrogenase (*hya* operon) and formate dehydrogenase (*fdhF*). At least some of these regulatory effects may reflect inactivation of FNR by NO (Cruz-Ramos *et al.*, 2002; Justino *et al.*, 2005; Pullan *et al.*, 2007), or regulation by the nitrate-sensing two-component systems NarXL and NarQP (Constantinidou *et al.*, 2006). Genes involved in glycolysis, gluconeogenesis, pyruvate metabolism [*eno*, *pykF*, *pgi*, *fba*, *gapA*, *glk*, *pgk*, *ldhA*, *maeA* (*sfcA*)], the pentose phosphate pathway (*talA*, *tktb*, *pgl*) and the metabolism of sugars (fructose, sucrose and mannose) showed decreased expression. Iron transport genes (*feoAB*) and some stress response genes (*clpB*, *dnaJ*, *dnaK*, *dps*) also showed reduced expression. Fig. S1(a) provides an overview of the differentially expressed genes based on their occurrence in pathways, and Fig. S1(b) provides an overview based on functional categories.

It is known that the citric acid cycle is repressed in *E. coli* grown anaerobically in nitrate with glucose as the carbon source (Prohl *et al.*, 1998). Under anaerobic conditions with glucose and nitrate, ArcA represses operons encoding α-ketoglutarate dehydrogenase and succinate dehydrogenase, thus preventing complete oxidation of glucose to carbon dioxide (Prohl *et al.*, 1998). However, our RNA-seq data for the 3X mutant show increases in the transcript levels of the genes encoding members of both these enzyme complexes as well as various other genes involved in the intact aerobic TCA cycle. This suggests that nitrate (and possibly NO generated by nitrate respiration) impacts the TCA cycle by allowing complete oxidation of acetyl-CoA and this may have a role to play in energy generation in the presence of NO. Our data are consistent with a recent finding that the TCA cycle is necessary for UPEC fitness *in vivo* (Alteri *et al.*, 2009), conditions in which UPEC is known to be exposed to NO (Lundberg *et al.*, 1996; Mysorekar *et al.*, 2002; Poljakovic *et al.*, 2001) as well as nitrate (Green *et al.*, 1981; Radomski *et al.*, 1978).

Numerous studies indicate that iNOS expression levels increase and NO is released in the urinary tract during bacterial infection (Kaboré *et al.*, 1999; Lundberg *et al.*, 1996; Smith *et al.*, 1996; Wheeler *et al.*, 1997). Also, dietary nitrate is excreted into urine where UPEC can potentially use it for respiration, generating NO as a byproduct (Corker & Poole, 2003; Ji & Hollocher, 1988; Lidder & Webb, 2013). Although the role of NO is presumably antimicrobial, the pathogen may use host NO (as well as NO derived from nitrate respiration) as a signal to induce the expression of virulence genes. We make this suggestion based on the observation that virulence-related genes are upregulated in UPEC cultures exposed to nitrate and/or to NO produced by nitrate respiration (Fig. S1c). The secreted autotransporter toxin (Sat) is a serine protease that causes cytoplasmic vacuolation and histological damage in urinary-tract-derived epithelial cells (Guyer *et al.*, 2002). The flagellar gene *fliC* contributes to fitness of UPEC and enhances its pathogenesis (Lane *et al.*, 2005), and other genes involved in flagellar assembly (*fliD*, *fliS*, *fliR*, *motAB* and *flgM*) were upregulated in nitrate-grown cells. The fimbrial site-specific recombinases *fimB* and *fimE* can be associated with virulence, as they regulate type 1 fimbrial gene expression. Type 1 fimbriae are known to enhance *E. coli* virulence in the urinary tract (Bryan *et al.*, 2006; Connell *et al.*, 1996). Haemolysin (encoded by *hly* genes) is a cytotoxin for renal proximal tubular epithelial cells and a haemolysin-deficient CFT073 mutant demonstrates significantly reduced cytotoxicity (Mobley *et al.*, 1990). The sialic acid capsule proteins (encoded by *kps* genes), also known as K antigens, encapsulate bacteria so that they can evade unspecific host responses (Jann & Jann, 1992; Rowe *et al.*, 2000). BipA is identified as a virulence regulator in enteropathogenic *E. coli* that regulates several processes such as flagella-mediated motility, resistance to host defence peptides and group 2 capsule gene clusters (Farris *et al.*, 1998; Rowe *et al.*, 2000). The dipeptide binding protein DppA delivers dipeptides to its cognate ABC-type transporter proteins. As sugar sources such as glucose, maltose and lactose are rare in the urinary tract, it is suggested that dipeptides and certain amino acids such as d-serine are important sources of nutrients for UPEC (Haugen *et al.*, 2007). Upregulation of *dppA* is observed during UTI (Subashchandrabose *et al.*, 2014). A periplasmic osmoprotectant, ProV, is upregulated during UTI (Subashchandrabose *et al.*, 2014) and we observe upregulation during our growth conditions as well. Very often, pathogens experience increased osmotic pressure at the site of infection and hence acquire osmoprotectants from the environment (Lewis *et al.*, 2012). KpsMT, DppA and ProV are ABC-type transporters that, under appropriate conditions, become important for viability, virulence and pathogenicity (Davidson *et al.*, 2008). Upregulation of all these virulence-related genes suggests that, while NO provides a defence mechanism for the host and nitrate provides an electron acceptor for pathogens, either or both could also provide a useful signalling mechanism for pathogenic bacteria to induce virulence.

**Fig. 4.**
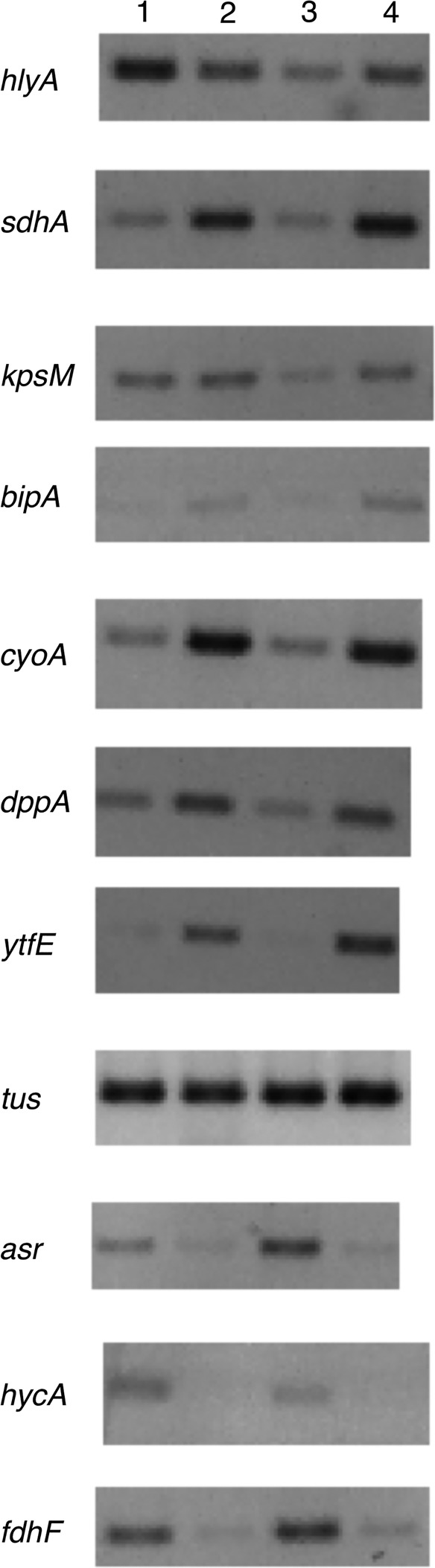
Measurement of gene expression by RT-PCR. Cultures of wild-type CFT073 (lanes 1 and 2) and the 3X mutant (lanes 3 and 4) were grown anaerobically in the absence (lanes 1 and 3) and presence (lanes 2 and 4) of nitrate. Genes were selected that were upregulated (*hlyA*, *sdhA*, *kpsM*, *bipA*, *cyoA*, *dppA* and *ytfE*) or downregulated (*asr*, *hycA* and *fdhF*) in response to nitrate in RNA-seq data. The *tus* gene served as a control. Total RNA was used for cDNA synthesis, and PCRs using cDNA as the template were primed with oligonucleotides directed against the selected genes.

Several genes were selected from the RNA-seq dataset for validation using RT-PCR. Expression of these genes was measured in both the wild-type strain and the 3X mutant grown anaerobically with nitrate (the same conditions used for RNA-seq). Of seven genes upregulated by nitrate according to RNA-seq data, six (*sdhA*, *kpsM*, *bipA*, *cyoA*, *dppA* and *ytfE*) were also upregulated in RT-PCR data, in both strains (Fig. 4). The seventh (*hlyA*) was upregulated only in the 3X mutant. Three genes that were downregulated by nitrate in RNA-seq data (*asr*, *hycA* and *fdhF*) were also downregulated according to RT-PCR (Fig. 4). On the basis of this selection of genes, we conclude that most changes observed in the transcriptome of the 3X mutant in response to nitrate are likely also to occur in the wild-type parent.

### The NsrR regulon of *E. coli* CFT073

As the *E. coli* CFT073 genome is ∼0.6 Mb larger than that of *E. coli* K-12, it is of interest to determine the extent to which regulatory networks of the two organisms differ. Thus, we used chromatin immunoprecipitation and DNA sequencing (ChIP-seq) to identify NsrR binding sites in the *E. coli* CFT073 genome. Cultures expressing 3Xflag-tagged NsrR were grown aerobically. After ChIP, libraries constructed from precipitated DNAs were sequenced using the Illumina Miseq platform. The peak finding algorithm MACS2 was used to identify putative NsrR binding sites, with a false discovery rate of 0.01. Ninety-four significant peaks [ − log_10_(*P*-value)>10 with fold enrichment greater than 2] were identified in at least two of the three biological replicates. In total, 52 % of the binding sites (49 of 94) in *E. coli* CFT073 were located in putative promoter regions (within 350 bp of the start codon) and the remaining 48 % were found either within coding regions or between the coding regions of convergent genes. These potentially functional 49 NsrR binding sites are shown in Table 2.

**Table 2.** NsrR binding sites in the *E. coli* CFT073 genome

Coordinate^*^	− log_10_(*P-*value)†	Fold enrichment‡	Flanking genes§	Distance from summit to start codon^∥^	Possible NsrR site¶	Sequence	PatSer score	PatSer ln(*P*-value)^#^
1890380	503.52	19.16	*grxD* ( < ) *mepH* (>)	− 138 (*grxD*)	*grxD* (74)	AAATGTTATTT	7	− 8.98
					*grxD* (121)	TTGTTGCATTT	7.19	− 9.15
					*grxD* (111)	AAAATACGTTT	5.44	− 7.45
3130600	523.77	16.18	*hycB* ( < ) *hycA* ( < )	− 59 (*hycB*)	*hycB* (54)	AAATGTCATTT	7.6	− 9.67
3645158	287.94	11.46	*folB* ( < ) *plsY* (>)	28 (*folB*)				
967971	248.24	12.00	*hcp* ( < ) *ybjE* ( < )	− 22 (*hcp*)	*hcp* (15)	AAGTTATATTT	9.19	− 11.59
					*hcp* (27)	AACATGTATAT	8.78	− 11.14
					*hcp* (11)	AAGTTGCATTA	8.92	− 11.34
5049151	247.18	9.65	*ytfE* ( < ) *ytfF* ( < )	− 50 (*ytfE*)	*ytfE* (33)	AAGATGCATTT	10.92	− 15.25
					*ytfE* (45)	AAGATGCATTT	10.92	− 15.25
					*ytfE* (128)	CAGATTCAGTT	4.07	− 6.32
115339	218.24	10.60	*mutT* (>) *c0118* (>)	18 (*c0118*)	*c0118* (77)	CATTTGCATAT	3.86	− 6.16
					*c0118* (6)	AAGGTGCAGTT	7.70	− 9.79
227553	163.73	9.01	*c0233* ( < ) *yaeF* ( < )	2 (*c0233*)	*c0233* (29)	AAGTTTTACTT	7.38	− 9.39
					*c0233* (17)	AACATTCATTT	9.43	− 12.21
					*c0233* (1)	AAGGTGCAGTT	7.70	− 9.79
4375989	117.47	5.31	*dgoK* ( < ) *dgoR* ( < )	222 (*dgoK*)				
3967122	116.05	5.92	*yhgF* (>) *feoAB*(>)	95 (*feoA*)	*feoA* (38)			
2314458	88.47	6.45	*c2470* ( < ) *c2471* (>)	− 66 (*c2471*)	*c2471* (122)	ATGTGATATTT	5.63	− 7.62
					*c2471* (84)	AAGTTTCATGT	7.45	− 9.49
					*c2471* (72)	TTGATGTTTTT	3.92	− 6.21
					*c2471* (56)	AGCTTGTATTT	4.55	− 6.69
4399249	72.9	4.67	*yieH* (>) *cbrB* (>)	4 (*yieI*)	*yieI* (18)	TACTTACCTTT	3.94	− 6.22
4224075	72.8	4.56	*waaH* ( < ) *tdh* ( < )	191 (*waaH*)				
698961	54.72	4.83	*ccrB* ( < ) *ybeM* (>)	345 (*ybeM*)				
4883923	49.17	3.65	*phnC* ( < ) *phnB* ( < )	274 (*phnC*)				
3639613	48.43	3.91	*ygiF* ( < ) *c3803* ( < )	232 (*ygiF*)				
2654737	48.2	4.66	*arnC* (>) *arnA* (>)	316 (*arnA*)				
4037958	46.57	3.97	*livJ* ( < ) *rpoH* ( < )	250 (*livJ*)				
4840905	45.39	3.67	*acs* ( < ) *c5065-nrfA* (>)	− 25 (*c5065*)	*c5065* (94)	AACATGCAGTT	8.12	− 10.23
					*c5065* (42)	AAGTGGTATTT	8.71	− 11.03
					*c5065* (31)	TACATGCACTT	6.82	− 8.79
					*c5065* (4)	ACATTCATAGT	5.41	− 7.42
796704	44.53	4.08	*cydB* (>) *c0813* ( < ) *ybgE* (>)	177 (*c0813*)				
3760376	34.92	3.61	*hflB* ( < ) *c3935* ( < )	152 (*hflB*)				
4476183	34.04	2.95	*wecE* (>)*c4712* ( < ) *wzxE* (>)	− 259 (*wzxE*)				
2350649	32.09	3.82	*c2513* ( < ) *c2514* (>)	200 (*c2514*)				
2948718	32.08	3.79	*glyA* ( < ) *hmp* (>)	34 (*hmp*)	*hmp* (44)	AAGATGCATTT	10.92	− 15.25
					*hmp* (19)	AAGATGCAAAA	5.00	− 7.08
4708187	28.99	3.45	*thrT* (>) *tufB* (>)	− 5 (*tufB*)				
2694874	27.98	3.58	*yfbT* ( < ) *yfbU* ( < )	− 281 (*yfbT*)				
1088139	27.33	2.24	*yccM* ( < ) *torS* ( < )	− 36 (*yccM*)	*yccM* (24)	AAGTTGCATAC	6.86	− 8.83
					*yccM* (36)	TAGTGGCATTT	7.63	− 9.69
					*yccM* (50)	TAGTTGTTCTT	3.97	− 6.25
4569962	26.65	3.45	*c4805* (>) *yihF* (>)	314 (*yihF*)				
628066	24.07	3.35	*ydfM* (>) *c0650* (>)	− 52 (*c0650*)	*c0650* (50)	AAGATGTATCG	3.95	− 6.23
4468281	23.37	2.71	*c4703* (>) *rfe* (>)	201 (*rfe*)				
4342139	23.29	2.93	*c4579* ( < ) *c4580* (>)	123 (*c4580*)				
2639159	23.00	3.28	*glpT* ( < ) *glpA* (>)	− 41 (*glpA*)	*glpA* (93)	ACGTTTCACTT	4.95	− 7.05
					*glpA* (50)	AACATGAATTG	4.74	− 6.86
4792020	22.78	2.78	*zur* ( < ) *yjbN* (>)	− 169 (*yjbN*)				
3999621	21.56	3.05	*c4214* ( < ) *glgP* ( < )	3 (*c4214*)	*c4214* (5)	AAGGTATAAAT	4.35	− 6.54
					*c4214* (69)	AAGTTATATCT	5.80	− 7.79
5208290	20.47	2.75	*deoA* (>) *deoB* (>)	18 (*deoB*)				
3131567	17.96	2.67	*hycA* ( < ) *hypAB* (>)	− 126 (*hypB*)	*hypB* (167)	CGAGGTGCAGT	4.13	− 6.37
4704549	17.63	2.75	*rrfB* (>) *murB* (>)	244 (*murB*)				
986391	16.91	2.87	*trxB* ( < ) *lrp* (>)	− 92 (*trxB*)	*trxB* (32)	TACTTAAATTT	4.92	− 7.01
					*trxB* (100)	ATGTTGTACTA	4.41	− 6.58
					*trxB* (112)	AACATCGATTT	4.90	− 7.00
4961829	15.97	2.80	*c5205* ( < ) *c5206* ( < )	4 (*c5205*)	*c5205* (7)	AACAGGTATTA	6.29	− 8.24
3335185	15.31	2.44	*recJ* ( < ) *dsbC* ( < )	− 106 (*recJ*)				
240582	15.04	2.73	*aspU* (>) *dkgB* (>)	14 (*dkgB*)	*dkgB* (18)	AAATAGCATTA	4.63	− 6.76
					*dkgB* (6)	AAGAGGCATAT	8.32	− 10.55
3016197	14.59	2.70	*recN* (>) *bamE* (>)	6 (*bamE*)	*c3139* (65)	AAGGTCTATTA	5.21	− 7.24
					*c3139* (46)	ATATTACAGAT	3.74	− 6.06
4616876	14.49	2.17	*rhaB* ( < ) *c4854* (>)	229 (*rhaB*)				
2522542	14.14	2.67	*yohJ* (>) *yohK* (>)	− 172 (*yohK*)				
3786570	14.05	2.36	*arcB* ( < ) *yhcC* ( < )	− 53 (*arcB*)				
3391606	13.75	2.47	*yggS* (>) *yggT* (>)	− 159 (*yggT*)	*yggT* (172)	TACAGCCATTT	4.94	− 7.03
2961332	13.69	2.64	*pgpC* ( < ) *c3084* ( < )	64 (*pgpC*)				
3795223	13.68	2.25	*nanK* ( < ) *nanE* ( < )	322 (*nanK*)				
5139372	12.82	2.57	*fimI* (>) *fimC* (>)	337 (*fimC*)				
4779126	11.28	2.07	*malF* ( < ) *malE* ( < )	− 142 (*malF*)	*malF* (82)	ACATACGTTTC	3.98	− 6.26
					*malF* (163)	AAGATGCACAG	>5.00	− 7.08

* Genomic location of the summit of ChIP-seq peak.

†  − log_10_(*P*-value) of each peak called by MACS2.

‡ Fold enrichment of each peak calculated by MACS2.

§ The genes flanking the ChIP-seq summit.

∥ The distance between the peak summit and start codon of the nearest downstream gene.

¶ Possible NsrR binding motifs identified by PatSer (Bailey & Elkan, 1994), and the distance from the motif to the start codon of the gene. Only sites upstream of start codons are shown.

# ln(*P*-value) associated with the PatSer score for each predicted NsrR site.

The presence of promoter-associated NsrR binding sites identifies target genes that potentially belong to the NsrR regulon. Of these promoters bound by NsrR *in vivo* (Table 2), 19 (*grxD*, *hypA*, *ytfE*, *ygiG*/*folB*, *hmp*, *ybjW*/*hcp*, *feoA*, *ybeM*, *yihF*, *yccM*, *yibD*/*waaH*, *yieI*, *yohK*, *ygiF*, *trxB*, *yggS*/*yggT*, *dgoK*, *rfe*, *yfhB*/*pgpC*) were identified in a previous ChIP-chip analysis of NsrR binding sites in *E. coli* K-12 (Partridge *et al.*, 2009). Twenty of the remaining sites are associated with genes (*hycB*, *phnC*, *arnA*, *livJ*, *wzxE*, *tufB*, *yfbT*, *glpA*, *yjbN*, *deoB*, *murB*, *recJ*, *dkgB*, *c3139*, *rhaB*, *arcB*, *c3976*/*nanK*, *fimC*, *c3934*/*hflB* and *malF*) that have homologues in *E. coli* K-12, and 10 (*c0118*, *c0233*, *c2471*, *c5065*, *c0813*, *c2514*, *c0650*, *c4580*, *c4214* and *c5205*) are specific to *E. coli* CFT073.

In *E. coli* K-12, the *nrfA* promoter is bound by NsrR (Partridge *et al.*, 2009) and is repressed by NsrR according to microarray and reporter fusion data (Filenko *et al.*, 2007). In our ChIP-seq data, NsrR binding was also detected upstream of the transcription unit that includes *nrfA*. In strain CFT073, an additional gene upstream of *nrfA* (*c5065*) is predicted to be co-expressed with *nrfABCD*. The *c5065* gene encodes a small protein of 65 aa residues. We have confirmed the sequence of this reading frame in the CFT073 genome. The genome location and expression pattern of the *c5065* gene suggest that its product may have a role in the response to NO stress in CFT073.

Nineteen of the 49 potential NsrR targets show differential expression with a fold change greater than 1.5 for the CFT073 3X mutant strain in the presence of a physiological source of NO (Table 3). The *hycB*, *c0118*, *feoA*, *ybeM*, *c5065*, *c0813*, *c2514*, *glpA*, *deoB*, *hypAB*, *yohJK* and *yfhB*/*pgpC* genes were downregulated, among which *hycB*, *c0118*, *c5065*, *c0813*, *c2514*, *glpA* and *deoB* are newly detected potential NsrR targets in CFT073. The *grxD*, *folB*, *ybjW*/*hcp*, *ytfE*, *yjbN*, *trxB* and *c5205* genes were upregulated and *c5205* and *yjbN* are potential NsrR targets newly detected in CFT073. The *glpA* gene, which encodes anaerobic glycerol-3-phosphate dehydrogenase subunit A, is downregulated in UPEC strain UTI89 exposed to acidified nitrite (Bower *et al.*, 2009). The *livJ* gene, which encodes a periplasmic Leu/Ile/Val-binding protein, is upregulated during *in vitro* growth in human urine (Snyder *et al.*, 2004). The *fimC* gene encoding type 1 fimbriae is upregulated *in vivo* during UTI (Snyder *et al.*, 2004). The *rfe* gene was upregulated *in vivo* compared with growth in human urine *in vitro* (Hagan *et al.*, 2010). The *E. coli* K-12 homologue of *yfbT* is upregulated in the presence of a source of NO (Hyduke *et al.*, 2007). In *E. coli* K-12, the expression of *arcB* and *malF* was increased and decreased, respectively, after treatment with NO (Hyduke *et al.*, 2007), and *phnC* was upregulated by treatment with 1 mM *S*-nitrosoglutathione or acidified nitrite (Mukhopadhyay *et al.*, 2004).

**Table 3.** NsrR binding sites associated with genes that are nitrate-responsive in RNA-seq data

Coordinate^*^	− log_10_(*P*-value)†	Fold enrichment‡	Flanking genes§	Distance from summit to start codon∥	Fold change after nitrate treatment¶	PPEE^#^
1890380	503.52	19.16	*grxD* ( < ) *mepH* (>)	− 138 (*grxD*)	1.59	0.005
3130600	523.77	16.18	*hycB* ( < ) *hycA* ( < )	− 59 (*hycB*)	0.01	0
3645158	287.94	11.46	*folB* ( < ) *plsY* (>)	28 (*folB*)	1.59	0.01
967971	248.24	12.00	*hcp* ( < ) *ybjE* ( < )	− 22 (*hcp*)	43.72	0
5049151	247.18	9.65	*ytfE* ( < ) *ytfF* ( < )	− 50 (*ytfE*)	69.63	0
115339	218.24	10.60	*mutT* (>) *c0118* (>)	18 (*c0118*)	0.36	2.8 × 10^− 7^
3967122	116.05	5.92	*yhgF* (>) *feoAB* (>)	95 (*feoA*)	0.18	0
698961	54.72	4.83	*ccrB* ( < ) *ybeM* (>)	345 (*ybeM*)	0.30	0
4840905	45.39	3.67	*acs* ( < ) *c5065-nrfA* (>)	− 25 (*c5065*)	0.57	0.002
796704	44.53	4.08	*c0813* ( < ) *ybgE* (>)	177 (*c0813*)	0.62	1.84 × 10^− 11^
2350649	32.09	3.82	*c2513* ( < ) *c2514* (>)	200 (*c2514*)	0.41	0
2639159	23	3.28	*glpT* ( < ) *glpA* (>)	− 41 (*glpA*)	0.23	0
4792020	22.78	2.78	*zur* ( < ) *yjbN* (>)	− 169 (*yjbN*)	2.91	0.01
5208290	20.47	2.75	*deoA* (>) *deoB* (>)	18 (*deoB*)	0.28	0
3131567	17.96	2.67	*hycA* ( < ) *hypAB* (>)	− 126 (*hypB*)	0.05	0.05
986391	16.91	2.87	*trxB* ( < ) *lrp* (>)	− 92 (*trxB*)	1.71	0
4961829	15.97	2.80	*c5205* ( < ) *c5206* ( < )	4 (*c5205*)	1.90	0.001
2522542	14.14	2.67	*yohJ* (>) *yohK* (>)	− 172 (*yohK*)	0.06	0
2961332	13.69	2.64	*pgpC* ( < ) *c3084* ( < )	64 (*pgpC*)	0.34	0

* Genomic location of the summit of ChIP-seq peak.

†  − log_10_(*P*-value) of each peak called by MACS2.

‡ Fold enrichment of each peak calculated by MACS2.

§ The genes flanking the ChIP-seq summit.

∥ The distance between the peak summit and start codon of the nearest downstream gene.

¶ Posterior fold change (the fold change computed from normalized data) calculated by EBSeq, shown for the predicted NsrR target.

# Posterior probability that a gene/transcript is not equally expressed under two conditions, as estimated by EBSeq.

### Computational analysis of NsrR binding sites in CFT073

**Fig. 5.**
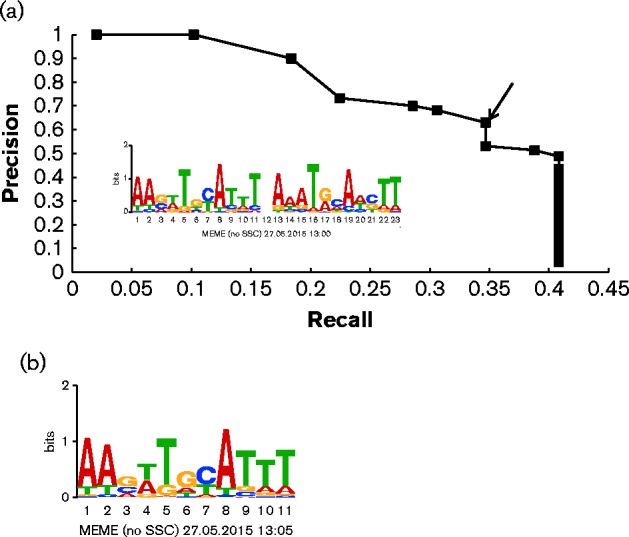
Computational prediction of NsrR binding sites. (a) Precision–recall curve used to determine the prediction threshold of NsrR binding sites. The precision and recall values were determined for many ln(*P*-value) thresholds using the PatSer algorithm and the optimal value ( − 14.28) is identified by the arrow. The inset shows the NsrR position weight matrix with inverted repeats constructed from the NsrR ChIP-seq sequences. (b) NsrR position weight matrix from NsrR ChIP-seq peak sequences. The height (*y*-axis) of the letters represents the degree of conservation at that position within the aligned sequences set (in bits), with perfect conservation being 2 bits. The *x*-axis shows the position of each base (1–11) starting at the 5′ end of the motif.

The 49 peaks located in putative regulatory regions were used to construct a PSWM for NsrR binding sites in the CFT073 genome. Two hundred base pairs centred on the nucleotide with the largest tag density within each of the peaks was analysed (Myers *et al.*, 2013). The sequence of NsrR in CFT073 is identical to that in *E. coli* K-12, and evidence from previous studies suggests that NsrR binding sites have two copies of an 11 bp motif arranged as an inverted repeat with 1 bp spacing (Partridge *et al.*, 2009). So, we first used MEME to identify over-represented palindromic sequences with the parameters ‘-mod zoops -nmotifs 1 -minw 23 -maxw 23 -revcomp–pal’ to see if the same motif could be retrieved. Motifs matching the search criteria could be found in 20 of the 49 peak regions. As expected, the predicted NsrR binding site in CFT073 is similar to that for *E. coli* K-12 (Fig. 5). A precision–recall curve (see Methods) was constructed using the NsrR PSWM with two inverted repeats and searching throughout the genome of CFT073 to determine the optimal threshold for predicting high-quality NsrR binding sites. Using an ln(*P*-value) of − 14.28 as the cut-off, where we had both relatively high precision and recall, there were 27 predicted NsrR binding sites with the 11–1–11 inverted repeat (palindrome) motif in the CFT073 genome (Table 4). Four of these predicted targets were not detected by the ChIP-seq data (*tehA*, *yeaR*, *yhiX* and *ygbA*). Among them, *yeaR* and *ygbA* are known to be regulated by NsrR (Bodenmiller & Spiro, 2006; Lin *et al.*, 2007), and the *ygbA* promoter was reported to be bound by NsrR in *E. coli* K-12 according to previous ChIP-chip data (Partridge *et al.*, 2009). Likewise, *tehA* was implicated as an NsrR target in *E. coli* K-12 by the same ChIP-chip data and by repressor titration (Bodenmiller & Spiro, 2006), and it was shown to be upregulated in the urinary tract in an asymptomatic bacteriuria strain of *E. coli* (Roos & Klemm, 2006). By contrast, reporter fusion data suggest that *tehA* is not regulated by NsrR (Bodenmiller & Spiro, 2006); conflicting reports may reflect differences in growth conditions or genetic background. Minimally, we can conclude that *yeaR* and *ygbA* are probably false negatives in our ChIP-seq data. The *gadX* (*yhiX*) gene was reported to be induced by NO through an indirect NsrR-dependent mechanism in *E. coli* O157 : H7 (Branchu *et al.*, 2014), but the presence of an NsrR binding site upstream of *gadX* may indicate a direct regulatory mechanism.

**Table 4.** NsrR binding sites with 11–1–11 inverted repeats in the *E. coli* CFT073 genome

Peak centre^*^	Downstream gene†	ChIP-seq‡	PatSer ln(*P*-value)§	Motif sequence∥
5049128	*ytfE*	+	− 25.13	AAGATGCATTTAAAATGCATCTT
967958	*hcp*	+	− 23.87	AAGTTATATTTAATATACATGTT
115376	*c0118*	+	− 22.88	AAGTTTTACTTCAAATGAATGTT
227478	*c0233*	+	− 22.88	AAGTTTTACTTCAAATGAATGTT
4569952	*yihF*	+	− 21.71	AAATTGTATTTGATGTGGATGTT
2948634	*hmp*	+	− 18.22	TTGATGTATCTCAAATGCATCTT
2654713	*arnA*	+	− 17.73	GAGGTGCATTTAATCTGCATGGT
1088121	*yccM*	+	− 17.63	TAGTGGCATTTGGTATGCAACTT
3999611	*c4214*	+	− 17.62	AAATTGAATTTCATTTATACCTT
4104796	*yhiX*	−	− 17.62	AAGATATATGTTATATGAATGTT
2037249	*yeaR*	−	− 17.05	AAATGGTATTTAAAATGCAAATT
1890357	*grxD*	+	− 16.81	TTGTTGCATTTCAAATATTCGTT
3130577	*hycB*	+	− 16.79	AAATGACATTTCATCGGCATGTT
1693010	*tehA*	−	− 16.74	AAAGTATATTTGAAATGCATTTT
3137982	*ygbA*	−	− 16.65	AAGGTGCATTTATATTACAACTT
3994013	*c4208*	−	− 16.45	AAAGTTTATTTATACTGAATGTT
4840882	*c5065*	+	− 16.32	TAAGTGCATGTAAAATACCACTT
4342117	*c4580*	+	− 16.16	AAGTTGCATTTTATCTGCACCGG
986393	*trxB*	+	− 16.15	ATGTTGTACTAAAAATCGATGTT
1920754	*ydiC*	−	− 15.97	AAGTTGCATTGAAAATGACTATT
3391575	*yggS*	+	− 15.94	AAGTTGCACGCCAAATGGCTGTA
1651193	*c1819*	−	− 14.83	ATATTACATTGGATATGAATGTA
460167	*c0470*	−	− 14.57	TAATTGCATATTAAAAATATGTT
4399231	*yieI*	+	− 14.5	AAAGGGAGTTTGATATGTCTGTT
3645157	*ygiG*	+	− 14.42	ATATTGTATTTATAGAGCAACTT
371521	*c0392*	−	− 14.31	TAGTTTCATTATATATGTCTGAT
1830291	*ynfL*	−	− 14.28	AAGATGTTTTAAATATGAATCTT

* Sites were identified using PatSer and a precision–recall curve was determined based on an ln(*P*-value) threshold of − 14.28. The coordinate of the centre of the predicted site is shown.

† The gene downstream of the predicted NsrR binding site.

‡ Presence (+) or absence ( − ) of an NsrR ChIP-seq peak at the location of each predicted NsrR binding site.

§ PatSer ln(*P*-value) of each predicted NsrR binding site (5′–3′).

∥ Sequence of each predicted NsrR binding site (5′–3′).

There is evidence that a single 11 bp motif can function as an NsrR-binding site in *E. coli* K-12 (Partridge *et al.*, 2009). So we combined the two halves of the 11–1–11 palindromic motif, and reconstructed a PSWM of 11 bp. The new 11 bp PSWM was used to scan the 49 200 bp sequences flanking all the peak regions using the *P*-value cut-off of 10^− 6^. In this analysis, 38 of 49 peaks had at least one single motif, and the updated sequence logo for the 11 bp motif is shown in Fig. 5(b).

## Discussion

Flavohaemoglobin (Hmp), flavorubredoxin (FlRd) and respiratory nitrite reductase (Nrf) have been extensively studied to understand their role in combating NO in *E. coli* and *Salmonella enterica* (Clarke *et al.*, 2008; Gardner, 2005; Gardner & Gardner, 2002; Gardner *et al.*, 2002; Gomes *et al.*, 2002; Mills *et al.*, 2008; Poock *et al.*, 2002; van Wonderen *et al.*, 2008). In *S. enterica*, it has been suggested that additional NO detoxification mechanisms are expressed in the absence of Hmp, and that the availability of different NO detoxification mechanisms under different environmental conditions is an important contributor to virulence (Mills *et al.*, 2008). Work in *E. coli* K-12 lacking the known NO detoxification mechanisms has also suggested the existence of an additional major pathway of NO metabolism (Vine & Cole, 2011b), which may be the same activity that we have observed in CFT073. In UPEC strains, the only system known to protect the pathogen from NO is Hmp (Svensson *et al.*, 2006, 2010). Competitive infection of UTI mouse models with wild-type and *hmp*-deleted UPEC strains showed a decreased ability of the mutant to infect (Svensson *et al.*, 2010). The roles of NrfA and FlRd have not been studied in the pathogen, but in this paper we show that FlRd is a major contributor to NO metabolism in UPEC, and that there is an additional NO-inducible activity yet to be identified. Our data suggest that the respiratory nitrite reductase Nrf makes only a minor contribution to NO metabolism.

Previous transcriptomic studies have suggested that UPEC experiences iron and oxygen limitation in the urinary tract (Hagan *et al.*, 2010; Snyder *et al.*, 2004). It has also been proposed that the ability of UPEC to adapt to low oxygen may be critical for successful bladder colonization during UTI (Subashchandrabose *et al.*, 2014). As UPEC is potentially exposed to NO (host derived and/or endogenously generated from nitrate respiration), nitrate and low oxygen *in vivo*, our choice of growth conditions for transcriptomics is relevant to the host environment. Our data (Fig. 4) suggest that responses to nitrate in the 3X mutant also occur in the wild-type parent. Nevertheless, the use of the 3X mutant may exacerbate responses to nitrate and NO compared with the parent strain. Our results have highlighted a group of interesting genes (including some that are virulence-associated) that we believe are good candidates for further investigation, including *in vivo* approaches. A disadvantage of the use of nitrate as a source of NO is that we cannot necessarily disentangle the effects of endogenously generated NO from direct effects due to nitrate. Thus, the changes observed in the transcriptome are likely to be mediated by nitrate sensing systems (NarXL/NarQP) in addition to those responsive to NO (principally NorR and NsrR). At least for those genes shared with *E. coli* K-12, we can use prior knowledge to infer some of the regulatory consequences of nitrate exposure. For example, upregulation of members of the NsrR regulon is strong evidence for the generation of physiologically significant concentrations of NO during nitrate respiration.

In this work, upregulation of genes involved in respiration and electron transport, along with genes associated with the TCA cycle, suggests that the pathogen uses these mechanisms to maximize energy generation during NO stress. Decreases in the levels of transcripts involved in glucose metabolism (glycolysis and gluconeogenesis) and upregulation of genes involved in dipeptide transport suggests that during NO stress, glucose may not be the energy source used by the pathogen. Differential expression of virulence-associated genes and genes on pathogenicity islands as a consequence of nitrate exposure suggest a role for nitrate and/or NO in pathogenesis. These experiments were performed with a mutant strain compromised in its ability to metabolize NO.

By ChIP-seq we identified NsrR binding sites in the CFT073 genome. Of 49 NsrR binding sites in promoter regions, 19 are associated with genes that were nitrate-responsive in the RNA-seq data. This discrepancy may reflect differences in the strains used, or the growth conditions used for the two experiments (aerobic growth for ChIP-seq, anaerobic growth for RNA-seq), although there is no published evidence to suggest that NsrR binding to DNA is sensitive to oxygen *in vivo*. Another possible explanation is that at some binding sites NsrR exerts weak or no regulation, as we have observed previously for *E. coli* K-12. As was the case for *E. coli* K-12 (Partridge *et al.*, 2009) around half of mapped sites were within coding regions or between convergently transcribed genes. Similar results have been obtained with other regulatory proteins, for example Fur (Seo *et al.*, 2014), and this is not surprising behaviour for a DNA-binding protein with a relaxed sequence specificity. We assume that most sites in this category have no biological function, although some may regulate the activity of promoters driving expression of small or anti-sense RNAs.

We found strong NsrR binding signals upstream of some hypothetical proteins of unknown function, some of them specific to CFT073 (meaning not present in *E. coli* K-12). Examples are c0118 and c0233, which are homologues of each other. Both c0118 and c0233 have two copies of a conserved helix–turn–helix domain that is often found in transposases and is likely to bind DNA. Both proteins are implicated as transposases or derivatives in the clusters of orthologous groups of proteins (COGs) database. Transposase genes are frequently associated with pathogenicity islands, and NsrR has been implicated in regulating pathogenicity island genes in *E. coli* O157 : H7 (Branchu *et al.*, 2014). Therefore, it would be interesting to study the function of c0118 and c0233 to see if they are related to the pathogenicity of CFT073, and to determine if NsrR is involved in the regulation of pathogenicity island genes.

Of the genes implicated as possible NsrR targets by ChIP-seq that were also differentially regulated in response to NO, two-thirds were downregulated in the presence of a source of NO. This behaviour is consistent with positive regulation by NsrR, as has been reported previously (Branchu *et al.*, 2014), or with indirect effects of NsrR. Some genes associated with NsrR binding sites were not differentially regulated in the RNA-seq experiment, which may indicate that these genes are subject to multiple regulatory mechanisms, such that regulation by NsrR is revealed only under specific growth conditions. An additional possibility is that there is a category of promoter that is bound by, but not regulated by, NsrR.

In conclusion, the response of UPEC strain CFT073 to NO overlaps substantially with that of *E. coli* K-12. In both cases, Hmp and FlRd provide the principal NO detoxification mechanisms, although there is evidence of additional activities yet to be identified. Anaerobic growth in the presence of nitrate (and therefore low concentrations of endogenously generated NO) causes a major reprogramming of the transcriptome. Major players in regulating differential gene expression under these conditions are likely to be NarXL, NarQP, FNR and NsrR. The NsrR regulon of CFT073 overlaps significantly with that of *E. coli* K-12, but our data also suggest that NsrR (and therefore NO) may regulate several CFT073 genes that do not have homologues in *E. coli* K-12.

## Supplementary Data

1150 Supplementary Data
