# Supplementary material for: Genome-wide analysis of the response to nitric oxide in uropathogenic Escherichia coli CFT073
Source: Microb Genom. 2015 Oct 13;1(4):e000031. doi: 10.1099/mgen.0.000031 (PMC5320621; doi:10.1099/mgen.0.000031)
Supplement: Supplementary file 1 — Supplementary Data [file mgen-01-31-s001.pdf]

Genome-wide analysis of the response to nitric oxide in uropathogenic *Escherichia coli* CFT073

Heer H. Mehta, Yuxuan Liu, Michael Q. Zhang and Stephen Spiro

Supplementary material

Table S1: List of strains and plasmids used in this work

Name	Genotype	Source or reference
UPEC strains:		
CFT073	Pyelonephritis isolate, P1 pap, P2 pap	Harry Mobley
UTD635	CFT073 *hmp*	This work
UTD680	CFT073 *norVW*	This work
UTD681	CFT073 *hmp norVW*	This work
UTD692 (3X)	CFT073 *hmp nrfA norVW*	This work
UTD717	CFT073 *hmp nrfA norVW nsrR*::*kan*	This work
UTD783	CFT073 *hmp nrfA norVW fnr*::*kan*	This work
UTD552	CFT073 *nsrR*::3X-Flag tag	This work
Plasmids:		
pCP20	FLP+, λ ci857+, λ p_R Rep^{ts}, Ap^R, Cm^R	Barry Wanner
pKD46	Red recombinase expression plasmid	Barry Wanner
pKD4, pKD3	Antibiotic cassette template for red recombinase mediated knockout	Barry Wanner

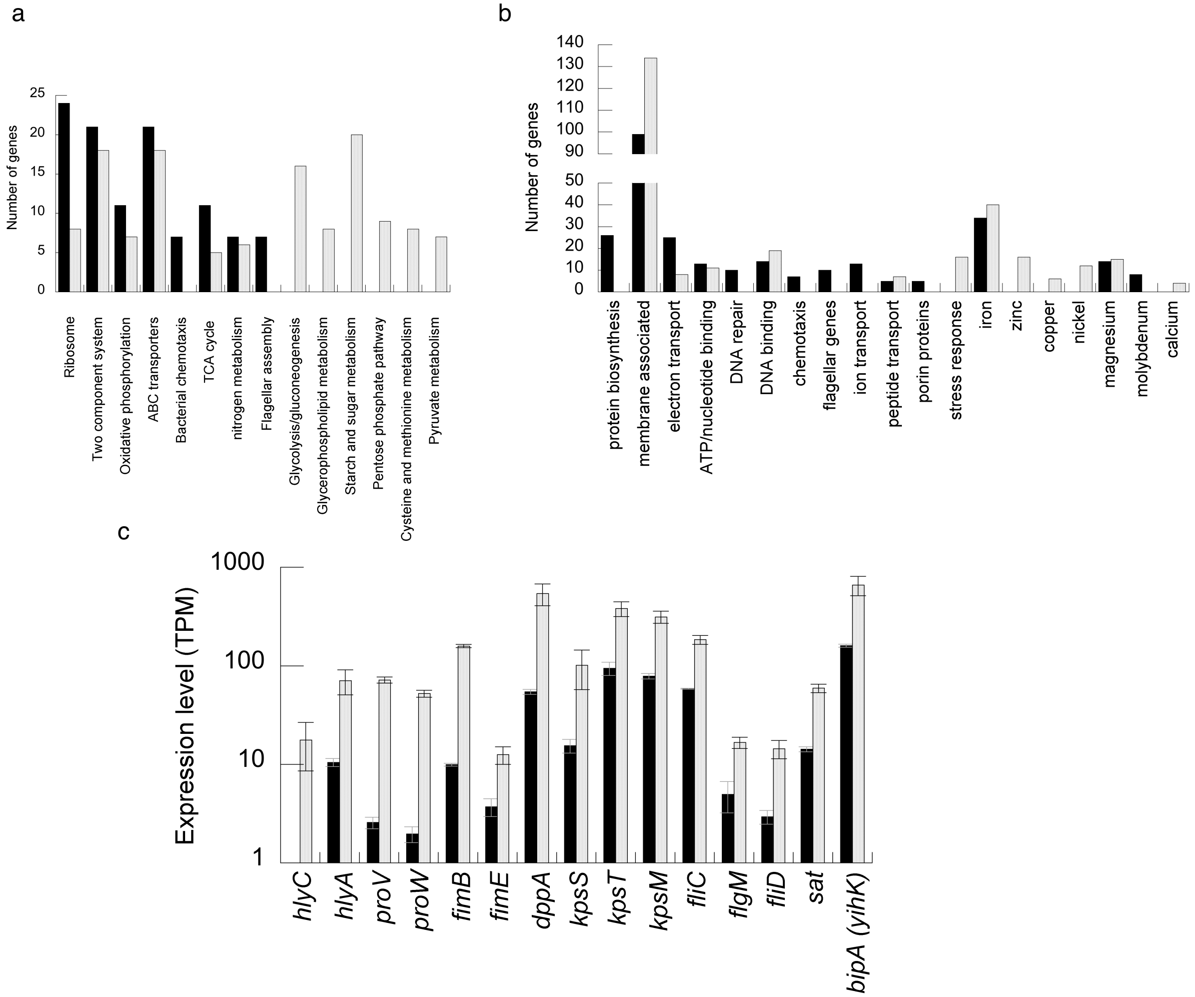

Figure S1. Functional categories and enriched pathways of differentially expressed genes. The number of genes that are up-regulated (black bars) and down-regulated (grey bars) in cultures exposed to nitrate, grouped by (a) occurrence in pathways, and (b) by functional categories. (c) Expression levels obtained from RNA-seq for virulence associated genes in cultures grown without (black bars) and with (grey bars) nitrate.
